# Comparative Genomic and Physiological Analysis against *Clostridium scindens* Reveals *Eubacterium* sp. c-25 as an Atypical Deoxycholic Acid Producer of the Human Gut Microbiota

**DOI:** 10.3390/microorganisms9112254

**Published:** 2021-10-29

**Authors:** Isaiah Song, Yasuhiro Gotoh, Yoshitoshi Ogura, Tetsuya Hayashi, Satoru Fukiya, Atsushi Yokota

**Affiliations:** 1 Laboratory of Microbial Physiology, Research Faculty of Agriculture, Hokkaido University, Sapporo 060-8589, Japan; isong@chem.agr.hokudai.ac.jp (I.S.); yokota@chem.agr.hokudai.ac.jp (A.Y.); 2 Department of Bacteriology, Faculty of Medical Sciences, Kyushu University, Fukuoka 812-8582, Japan; yasgotoh@bact.med.kyushu-u.ac.jp (Y.G.); thayash@bact.med.kyushu-u.ac.jp (T.H.); 3 Department of Infectious Medicine, Kurume University School of Medicine, Kurume 830-0011, Japan; y_ogura@med.kurume-u.ac.jp

**Keywords:** secondary bile acid, deoxycholic acid, *bai* operon, gut microbiome, *Clostridium scindens*

## Abstract

The human gut houses bile acid 7α-dehydroxylating bacteria that produce secondary bile acids such as deoxycholic acid (DCA) from host-derived bile acids through enzymes encoded by the *bai* operon. While recent metagenomic studies suggest that these bacteria are highly diverse and abundant, very few DCA producers have been identified. Here, we investigated the physiology and determined the complete genome sequence of *Eubacterium* sp. c-25, a DCA producer that was isolated from human feces in the 1980s. Culture experiments showed a preference for neutral to slightly alkaline pH in both growth and DCA production. Genomic analyses revealed that c-25 is phylogenetically distinct from known DCA producers and possesses a multi-cluster arrangement of predicted bile-acid inducible (*bai*) genes that is considerably different from the typical *bai* operon structure. This arrangement is also found in other intestinal bacterial species, possibly indicative of unconfirmed 7α-dehydroxylation capabilities. Functionality of the predicted *bai* genes was supported by the induced expression of *baiB*, *baiCD*, and *baiH* in the presence of cholic acid substrate. Taken together, *Eubacterium* sp. c-25 is an atypical DCA producer with a novel *bai* gene cluster structure that may represent an unexplored genotype of DCA producers in the human gut.

## 1. Introduction

Bile acids are cholesterol-derived compounds that are synthesized as primary bile acids by the liver and secreted into the gastrointestinal tract to aid in digestion of fat-soluble nutrients following a meal [1,2]. These primary bile acids circulate between the liver and gastrointestinal tract in a process known as enterohepatic circulation, with about 95% actively reabsorbed into circulation upon reaching the terminal ileum [3]. However, the remaining 5% continue into the colon and are subjected to gut microbiota-mediated biotransformation, including 7α/β-dehydroxylation of primary bile acids into what are known as secondary bile acids [2,3]. The two most abundant primary bile acids in humans are cholic acid (CA; 3α,7α,12α-trihydroxy-5β-cholan-24-oic acid) and chenodeoxycholic acid (CDCA; 3α,7α-dihydroxy-5β-cholan-24-oic acid), which are metabolized into the secondary bile acids deoxycholic acid (DCA; 3α,12α-dihydroxy-5β-cholan-24-oic acid) and lithocholic acid (3α-hydroxy-5β-cholan-24-oic acid), respectively. DCA is the most prevalent bile acid found in human fecal and cecal samples, making up roughly 34% of bile acids in both [3,4]. This is attributed to efficient passive reabsorption of DCA into the circulating bile acid pool due to its increased lipophilicity [5] as well as the human body’s inability to 7α-hydroxylate DCA back into its primary bile acid configuration. DCA has long been implicated in health issues such as liver and colon carcinogenesis [6,7,8,9] and cholesterol gallstone disease [10,11], but it has also been observed to enhance resistance to *Clostridioides difficile* colonization [12] and even suppress tumorigenesis in gallbladder cancer [13]. The holistic impact of DCA on human health and host-microbe interplay is not well-understood but warrants further investigation due to its biomedical importance.

**Figure 1:**
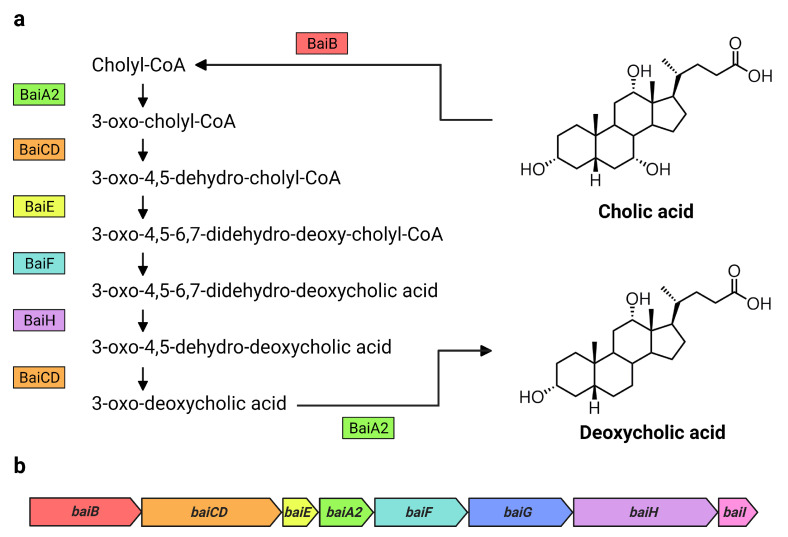
Bai proteins and *bai* genes involved in the formation of DCA from CA. Overview of (**a**) the 7α-dehydroxylation of CA to DCA including Bai proteins and intermediate products in *C. scindens* VPI 12708, as proposed by Funabashi et al. [14], and (**b**) the arrangement of the *bai* operon in *C. scindens* VPI 12708 and ATCC 35704^T^ [2]. Bai proteins are color coded with their respective genes in the *bai* operon. Figure created with BioRender.com.

7α-dehydroxylating bacteria possess a cluster of bile-acid inducible (*bai*) genes responsible for 7α-dehydroxylation of primary bile acids [3]. The core genes (*baiA2-I*) each encode a necessary enzyme in the multi-step 7α-dehydroxylation pathway and form a single operon [14] (Figure 1). While substantial amounts of DCA are produced solely by microbial biotransformation [3], the currently known DCA producers consist of a very small number of species belonging to the genus *Clostridium* and other closely related genera [2,15]. Their actual population numbers and diversity are unknown. It is estimated that the 7α-dehydroxylating pathway exists only in about 0.0001% of the colonic microbiota [3]. However, a recent large-scale analysis of public metagenome-assembled genome data suggests that up to almost 1% of intestinal bacteria may possess *bai* genes, and that these strains are taxonomically diverse [16,17]. In another study, a fecal cell count of *Clostridium scindens* [18], a DCA producer known to possess high 7α-dehydroxylation activity relative to other known DCA producers [19], revealed very low cell numbers (10^5.5^ cells/g of feces) and no significant correlation between *C. scindens* abundance and DCA levels in healthy Japanese subjects [20]. It is therefore necessary to further investigate the existence of other 7α-dehydroxylating bacteria and understand to what extent they contribute to DCA formation in humans.

In the early 1980s, Dr. Hirano’s laboratory published their discovery of 13 new 7α-dehydroxylating strains isolated from healthy adult fecal samples and wastewater [21]. Of these, three strains from adult fecal samples were not readily identifiable as *Clostridium* species, including a non-sporulating, Gram-positive “*Eubacterium lentum*-like” strain, “c-25” [21,22] (referred to as *Eubacterium* sp. c-25 in our study). This strain demonstrated the ability to 7α-dehydroxylate both CA and CDCA in vitro [21,22]. While the physiology and bile acid metabolism of c-25 were studied to an extent over the course of several years following its discovery [21,22,23,24,25], there are no recent reports studying this strain, and current mentions of the strain are almost nonexistent. As such, c-25 is poorly understood, especially in the context of our present-day knowledge of secondary bile acid metabolism. The lack of genomic and taxonomic information also limits our ability to compare the strain to other intestinal DCA producers.

The aim of this study was to expand upon our understanding of the previously described physiology of c-25 by gathering additional data supported by genomic information. As c-25 reportedly lacked the characteristic traits of *Clostridium* species [21], we believed it could be a taxonomically distinct, non-*Clostridium* intestinal DCA producer. We ran comparative tests of c-25 with two strains of *C. scindens*, a species which has been studied extensively as a representative intestinal DCA producer for years [3,18,26,27] and thus served as a baseline for our studies. The *C. scindens* strains used were: ATCC 35704^T^ [26,28] and G10, a rat cecal strain isolated in our previous study [29]. For our investigation, emphasis was placed on the conditions necessary for optimal growth and DCA production and the genetic differences between c-25 and other DCA producers, including genome-based phylogeny and expression of the *bai* gene cluster. Taken together, these data also give insight into intestinal conditions conducive for DCA formation, the unexplored diversity of DCA producers, and the genetic basis for 7α-dehydroxylation.

## 2. Materials and Methods

### 2.1. Bile Acids

The bile acids used in this study were purchased from the following companies: sodium salts of CA and DCA, Merck Sigma-Aldrich (Darmstadt, Germany); 7-oxo-deoxycholic acid (7-oxo-DCA; 3α,12α-dihydroxy-7-oxo-5β-cholan-24-oic acid), Steraloids, Inc. (Newport, RI, USA).

### 2.2. Bacterial Strains and Culture Conditions

*Eubacterium* sp. c-25 was kindly provided by Dr. Hiroshi Oda (Department of Bacteriology, Faculty of Medicine, Kagoshima University, Kagoshima, Japan) for our previous study [30]. *Clostridium scindens* ATCC 35704^T^ was provided by the RIKEN BRC through the National BioResource Project of the MEXT/AMED, Japan, under the name *Clostridium scindens* JCM 6567^T^, and *Clostridium scindens* G10 was isolated in our laboratory (Microbial Physiology, Hokkaido University) from rat cecal contents [29]. All strains were stored at −80 °C in 25% glycerol solution and were cultured anaerobically in Gifu Anaerobic Medium (GAM; Nissui Pharmaceutical Co. Ltd., Tokyo, Japan) at 37 °C. Freshly prepared GAM has an initial pH of approximately 7 and any necessary pH adjustments (i.e., pH 6, 8, and 9) were made using HCl or NaOH prior to autoclave sterilization. Dissolved oxygen was removed by leaving medium in a sealed pouch overnight with AnaeroPack oxygen-removal catalysts (Mitsubishi Gas Chemical Co., Inc., Tokyo, Japan). Initial pre-cultures were prepared by directly inoculating tubes of liquid GAM with cells from frozen stock cultures and incubating in an anaerobic chamber (Coy Laboratory Products Inc., Grass Lake, MI, USA) partially unsealed. Bacterial growth was measured as optical density at 660 nm (OD_660_) using the UV-1800 spectrophotometer (Shimadzu Corp., Kyoto, Japan). The initial pre-cultures were sub-cultured a minimum of one time, with the inoculum volume adjusted for an initial OD_660_ of 0.05 in subsequent cultures. All sub-cultures were prepared using source cultures at late-exponential to early stationary growth phase. Test cultures were incubated in butyl rubber-sealed Hungate screw-cap tubes in a 37 °C water bath after flushing the headspace with an anoxic gas mixture through the rubber stopper via 25G syringe tip. The same gas composition of N_2_, CO_2_, and H_2_ (8:1:1) was used for both the culture headspace and anaerobic chamber atmosphere. Bacterial growth in the test cultures was measured by OD_660_ using the SPECTRONIC 20D^+^ spectrophotometer (Thermo Spectronic, Rochester, NY, USA) over a period of 48 h. Bile acids were not added to pre-cultures, while test cultures were supplemented with CA to a concentration of 0.1 mM. As DCA was shown to have a strong inhibitory effect on tested secondary bile acid producers (data not shown), the initial CA substrate was limited to 0.1 mM to minimize this effect.

### 2.3. Quantification of Bile Acids in Bacterial Cultures by UPLC-ESI-MS/MS

For analysis of bile acid content in bacterial cultures, 100 µL of culture medium was collected per time point using syringes flushed with filter-sterilized anoxic gas. Samples were acidified with 20 µL of 3N HCl and stored at −30 °C until use. Five volumes of ethyl acetate were added to the acidified samples, which were then vortexed for 1 min and centrifuged at 18,000× *g* for 5 min to induce phase separation. The upper phase was collected and dried by centrifugal evaporation, re-suspended in methanol, and purified by gravity flow through Oasis HLB 1cc extraction cartridges (Waters Corp., Milford, MA, USA). Bile acids were eluted in 1 mL methanol, dried by centrifugal evaporation, and stored at −30 °C until further analysis.

To quantify the bile acids, the dried, purified samples were re-suspended in 1 mL of methanol, from which 200 µL was taken for bile acid quantification using the ACQUITY UPLC System (Waters Corp.) and Quattro Premier XE Mass Spectrometer (Waters Corp.) using an analytical method described in the literature [31] modified for one of our previous studies [29]. An ACQUITY UPLC BEH C18 column (Waters Corp.) was used for sample separation. Solvents A (80% (*v*/*v*) acetonitrile solution containing 10 mM ammonium acetate) and B (20% acetonitrile (*v*/*v*) solution containing 10 mM ammonium acetate) were used in a 2-minute, 50:50 isocratic flow method with a flow rate of 0.4 mL/min to separate CA, DCA, and 7-oxo-DCA, which were then quantified through selected ion recording. The *m*/*z* values scanned were 407.57 for trihydroxylated bile acids (CA), 391.60 for dihydroxylated bile acids (DCA), and 405.56 for dihydroxylated, mono-oxo-bile acids (7-oxo-DCA).

CA, DCA, and 7-oxo-DCA concentrations were calculated from the raw chromatogram data by comparison of peak area against standard curves of each bile acid constructed from two-fold serial dilutions within a concentration range of 0.195–6.25 μM. The concentrations were added together and interpreted as the total bile acid pool. Percent composition was then calculated based on the proportion of each bile acid relative to the total concentration of CA, DCA, and 7-oxo-DCA.

### 2.4. Scanning Electron Microscopy (SEM) Imaging

*Eubacterium* sp. c-25 cultures were grown in 8 mL of GAM for 6, 12, and 24 h, associated with mid-exponential, late-exponential, and stationary growth phase, respectively. Cells were harvested by centrifugation at 18,000× *g* for 5 min, washed with phosphate-buffered saline (PBS; 20 mM potassium phosphate buffer, 0.85% (*w*/*v*) NaCl, pH 7.4), and fixed onto a glass slide coated with poly-L-lysine with 2.5% (*v*/*v*) glutaraldehyde (TAAB Laboratories Equipment Ltd., Berkshire, UK) in PBS for one hour at room temperature. Slides were then washed with PBS and subjected to a secondary fixation step with 2% (*v*/*v*) osmium tetroxide in PBS for 30 min, again at room temperature. Following fixation, the cells were dehydrated by washing with a graded series of ethanol solutions (50%, 70%, 90%, 99.5% *v*/*v*) for 10 min intervals, with the highest concentration ethanol wash performed twice. The cells were then dried by critical point drying using liquid carbon dioxide (EM CPD300; Leica Microsystems, Wetzlar, Germany) and subsequently coated with a 12-nm layer of Au-Pd via ion sputtering (MSP-20-MT; Vacuum Device, Mito, Japan). Samples were visualized by scanning electron microscope (JSM-6301F; JEOL Ltd., Tokyo, Japan) at an acceleration voltage of 5 kV.

### 2.5. Complete Genome Sequencing of Eubacterium sp. c-25 and C. scindens G10

The genome sequences of *Eubacterium* sp. c-25 and C. *scindens* G10 were determined using a combination of Illumina MiSeq (Illumina, Inc., San Diego, CA, USA) and Nanopore MinION (Oxford Nanopore Technologies, Ltd., Oxford, UK) sequencing. For the first set of sequence data, *Eubacterium* sp. c-25 and *C. scindens* G10 genomic DNA (gDNA) was extracted from harvested cells from cultures grown in an anaerobic chamber until stationary phase. Using a previously described method [32], cells were subjected to enzymatic cell wall digestion by lysozyme at a final concentration of 15 mg/mL (FUJIFILM Wako Pure Chemical Corp., Osaka, Japan) and DNA extraction using the Isoplant II kit (Nippon Gene Co., Ltd., Tokyo, Japan). All gDNA extractions in this study were followed by quantification with the Qubit 3.0 Fluorometer (Thermo Fisher Scientific, Inc., Waltham, MA, USA). Short-read sequencing libraries were prepared with the NEBNext Ultra II FS DNA library preparation kit for Illumina (New England Biolabs, Inc., Ipswich, MA, USA) and then sequenced using Illumina MiSeq to obtain 301 bp paired-end reads.

High-molecular weight gDNA for Nanopore MinION sequencing was extracted from harvested *Eubacterium* sp. c-25 and *C. scindens* G10 cells in stationary phase by first subjecting them to enzymatic cell wall digestion at 37 °C with lysozyme (15 mg/mL, final concentration) for one hour followed by achromopeptidase (3 mg/mL, final concentration; FUJIFILM Wako Pure Chemical Corp.) for 30 min. This was followed by overnight incubation with Proteinase K (QIAGEN, Hilden, Germany) at 37 °C. The lysed samples were then treated with Tris-EDTA (TE)-saturated phenol and PCI (phenol, chloroform, and isoamyl alcohol at a 25:24:1 ratio) and stored overnight at 4 °C after RNase A (Nippon Gene) addition. Further purification was conducted using the Genomic-tip 20/G (QIAGEN) as per manufacturer’s instructions, with the exception of replacing vortex steps with gentle tube inversions to prevent shearing of gDNA. Long-read sequencing libraries were prepared with the Rapid Barcoding Kit (SQK-RBK004; Oxford Nanopore Technologies, Ltd.), sequenced using an R9.4.1 flow cell on the Oxford Nanopore MinION (Oxford Nanopore Technologies, Ltd.), and basecalled using Guppy GPU v3.4.5 (Oxford Nanopore Technologies, Ltd.).

Hybrid assembly of Illumina MiSeq and Nanopore MinION genome sequence data was conducted using Unicycler v0.4.7 [33,34]. Assembled genomes were then annotated using the DFAST pipeline [35], run in parallel with Prodigal [36] for structural annotation. The closed *Eubacterium* sp. c-25 and *C. scindens* G10 genomes were submitted to DDBJ under the accession numbers AP024845 and AP024846, respectively.

### 2.6. Bioinformatics Analyses

To identify phylogenetic relatives of *Eubacterium* sp. c-25, a BLASTn search for the c-25 16S rDNA sequence was conducted against the NCBI non-redundant Nucleotide database. Additionally, local BLASTn analysis of 16S rDNA sequence similarity across known DCA producers and other relevant strains was performed using GENETYX Ver.12 (Genetyx Corp., Tokyo, Japan). A Maximum Likelihood phylogenetic tree based on 16S rDNA sequences aligned using ClustalW [37] was constructed in MEGA X [38] using the Tamura–Nei model [39]. Robustness was assessed using 1000 bootstraps and other settings were left at default. Species-level taxonomic delineation was conducted by calculating average nucleotide identity (ANI) between genomes using the Orthologous Average Nucleotide Identity Tool (OAT) [40]. Information on all genomes used for bioinformatics analyses is listed in Table 1. OrthoFinder [41,42] was used to identify probable *bai* genes in *Eubacterium* sp. c-25 and in other potential secondary bile acid producers by virtue of its genome-wide orthologue inference algorithm. Identifiers for all predicted *bai* genes are listed in Table S1.

For comparative analysis of *bai* genes, local BLASTp was conducted using GENETYX Ver.12 to compare amino acid sequence similarity of *bai* gene products. Genomic locations of *bai* genes were determined using the genome visualization tool Artemis [43]. Promoter prediction within the *bai* gene cluster was performed using the BPROM program [44] offered by Softberry (http://www.softberry.com/; accessed on 7 September 2021).

In order to search for strains that could share a similar arrangement of *bai* genes with c-25, a Microbial Protein BLASTp search was conducted using c-25’s *baiB* protein sequence. The genomes of cultured and isolated strains possessing a candidate protein above a percent sequence identity threshold of 70% were acquired for further analyses. The metabolic gene cluster identification pipeline gutSMASH [45] was used to detect the presence of the *bai* operon in the candidate strains and perform preliminary arrangement prediction based on reference metabolic gene cluster databases.

### 2.7. Measurement of bai Gene Expression Using qRT-PCR

Expression of potential *bai* genes identified in *Eubacterium* sp. c-25 and *C. scindens* G10 was measured using real-time quantitative reverse transcription PCR (qRT-PCR) on the StepOnePlus Real-Time PCR System (Applied Biosystems, Waltham, MA, USA). Based on the structure of the predicted *bai* gene cluster in c-25, three genes were selected for measurement: *baiB*, *baiCD*, and *baiH*. Primers were constructed using the web-based Primer-BLAST [46] tool at default settings with strain-specific genomes as custom databases. Primer sequences are detailed in Table S2. All bacterial cultures were grown in GAM (pH 8) with or without 0.1 mM CA and sampled for analysis after 8 h of growth. Samples were washed with saline (0.85% [*w*/*v*] NaCl) and treated with RNAprotect Bacterial Reagent (QIAGEN), pelleted, and immediately frozen with liquid nitrogen for storage at −80 °C. Total RNA was extracted using a combination of enzymatic cell wall digestion and mechanical cell disruption as described in a separate study [47]. Synthesis of cDNA and analysis using qRT-PCR were also performed according to the methods and parameters outlined in the aforementioned study [47]. Gene expression was measured using the standard curve method, in which expression levels of reference housekeeping gene *recA* in serially diluted samples were used as a baseline for determining relative expression of the genes of interest, as conducted in the *bai* gene expression analysis in *C. scindens* ATCC 35704^T^ [26].

### 2.8. Statistical Tests

All statistical tests were conducted using R [48]. qRT-PCR data was first subjected to equal variance testing, and was analyzed using Welch’s *t*-test if the equal variance testing was significant (*p* < 0.05). Otherwise, the data was analyzed using Student’s *t*-test. All *t*-tests were one-tailed, as *bai* gene expression was expected to increase in G10 and c-25, as reported in *C. scindens* in the presence of CA [26]. In this context, we would interpret a decrease in expression as the practical equivalent of no significant increase, as either result is a strong indicator that the products of the genes in question are not directly involved in 7α-dehydroxylation.

## 3. Results

### 3.1. Morphology

**Figure 2:**
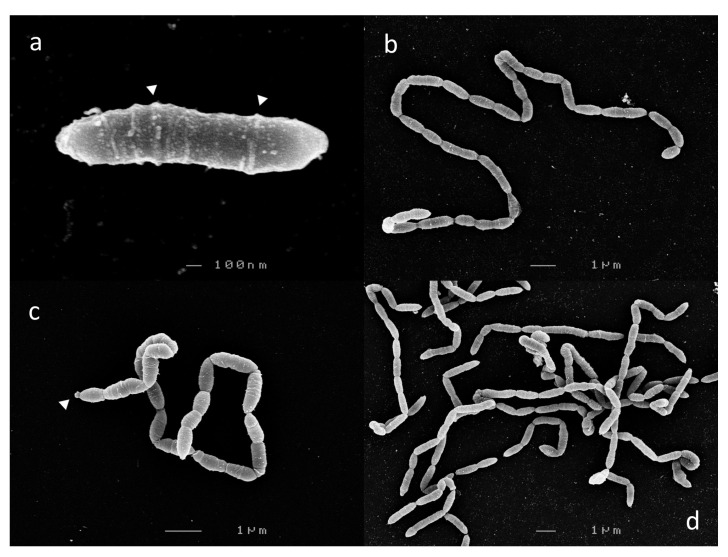
Scanning electron micrographs of *Eubacterium* sp. c-25. Observed morphological states: (**a**) single-cell, (**b**,**c**) filamentous, and (**d**) entangled filaments. Visible extracellular structures are indicated by white arrows. Images shown were taken after 6 h (**a**,**b**,**d**) or 12 h (**c**) of growth.

Cells from 6 h c-25 cultures, corresponding to mid-exponential growth phase, were harvested for SEM visualization. In a previous report, c-25 was observed to exist either as single, bacillus-shaped cells or in short chains of two to three cells [21]. However, we observed that c-25 cells formed long, end-to-end linked, non-branching filaments, with the single-cell morphology being exceedingly rare (Figure 2). Due to these structural traits, cell boundaries were sometimes difficult to delineate. Most segments were 1–2 µm in length, with some rare outliers reaching up to 8 µm.

Laterally arranged surface structures were found on virtually every cell, often forming a ring-like pattern (Figure 2a, arrows). Flagella and cillia were not visible. Large, roughly spherical structures were also found at the terminal ends of some cell chains (Figure 2c, arrow).

There were no noticeable differences in c-25 morphology in cells observed at 12 h (Figure 2c) and 24 h (not shown) time points corresponding to late-exponential and stationary growth phase, respectively.

### 3.2. Growth and Bile Acid Profile

The growth and DCA production of *Eubacterium* sp. c-25 were compared to *C. scindens* strains G10 and ATCC 35704^T^ in a series of culturing experiments. A point of interest is the ability of c-25 to grow and produce DCA in various pH conditions, as studies show that gastrointestinal pH is highly variable along the length of the intestines and is influenced by various physiological factors [49,50,51]. The composition of microbiotal communities is known to differ by gastrointestinal region based on the physiochemical properties of the local environment [52]. Additionally, the optimal pH for 7α-dehydroxylation enzymatic activity reported in early studies using washed whole cells was 7.3–7.7 for c-25 [22] and 7.0 for *C. scindens* strain VPI 12708 [53], warranting investigation into the effects of pH on DCA production in metabolically active in vitro cultures over time. To this end, medium pH was adjusted to an initial pH of 6, 7, 8, or 9 (±0.15) and pre-cultures that were grown at pH 7 were used for inoculation. Growth and bile acid production profile were measured over 48 h. The medium was not buffered because preliminary data showed noticeable decreases in the growth of c-25 when in the presence of several tested organic buffers (data not shown). For this reason, medium pH adjustment resulted in a transient initial pH that slowly decreased over time due to a combination of carbonic acid formation from aqueous carbon dioxide and acidic bacterial metabolites. Initial and final medium pH values were measured in each culture and can be found in Table S3.

The bile acids analyzed in each sample were CA, DCA, and 7-oxo-DCA, because these were the only bile acids detected during initial bile acid screening of CA-supplemented c-25 cultures against a panel of 31 bile acids (data not shown). 7-oxo-DCA is formed from CA through a reversible reaction catalyzed by 7α-hydroxysteroid dehydrogenases [3]. Accordingly, CA, DCA, and 7-oxo-DCA were interpreted as the total quantitatively relevant bile acid pool associated with CA metabolism.

**Figure 3:**
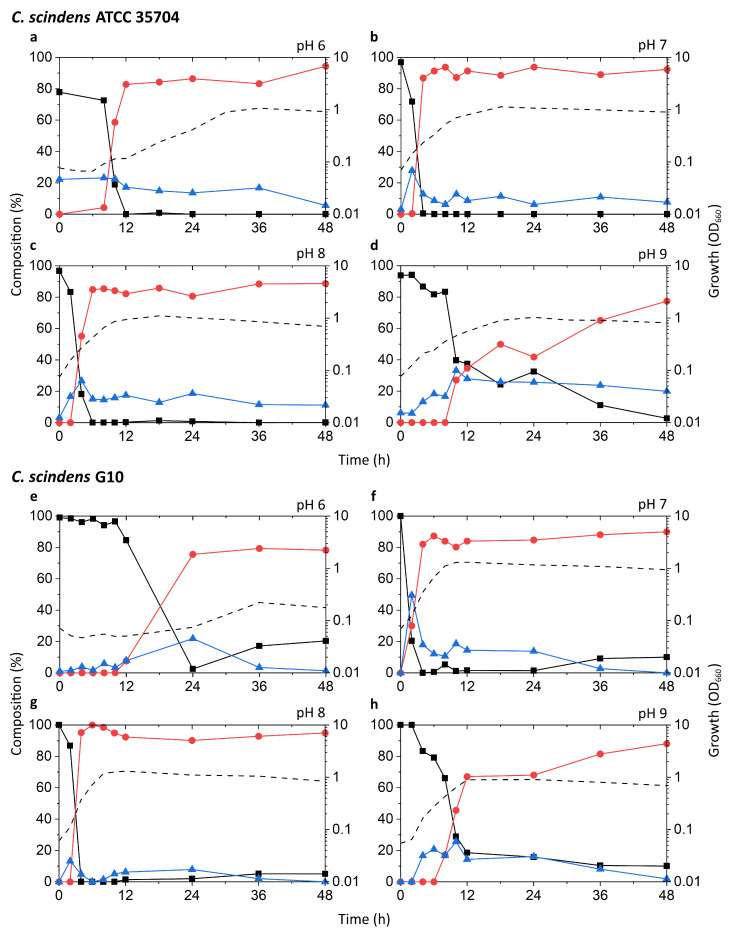

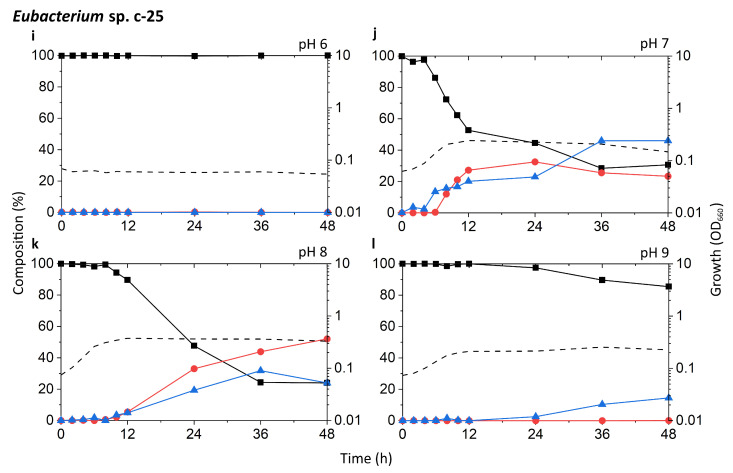
Time-course analysis of the growth and bile acid profile of DCA producers including *Eubacterium* sp. c-25 under different initial pH conditions. Tested strains are: (**a**–**d**) *C. scindens* ATCC 35704^T^, (**e**–**h**) *C. scindens* G10, and (**i**–**l**) *Eubacterium* sp. c-25. Growth was measured by OD_660_ (dotted line). The percent composition is calculated by the concentration of a given bile acid relative to the summed concentration of CA (black square), DCA (red circle), and 7-oxo-DCA (blue triangle). The average values of three independent experiments are shown.

Known DCA producers have been reported to possess varying degrees of 7α-dehydroxylation capabilities, with *C. scindens* exhibiting particularly high activity up to ~100-fold higher than other strains [19]. The results of our tests appear to be consistent with these reports (Figure 3a–h). The highest peak growth, along with the most rapid increase in DCA and decrease in CA, was observed at pH 7 and 8 in both G10 and ATCC 35704^T^. In these two pH conditions, relative levels of CA dropped rapidly within 2–6 h of incubation, coinciding with early to mid-exponential growth phase. This drop was accompanied by a proportionate increase in DCA, which plateaued at about 80–90% of the bile acid pool. 7-oxo-DCA was observed at a constant 5–10% of the bile acid pool following the initial spike in DCA. While growth and DCA formation were also observed at pH 6 and 9, the rate of growth was lower and CA-to-DCA conversion was delayed. Interestingly, DCA formation was more favorable at pH 9 than 6 for G10, while the opposite was true for ATCC 35704^T^. DCA formation was not inexorably linked to cell density, as pH 6 cultures of both *C. scindens* strains exhibited considerably high DCA formation at low OD_660_ in the early stages of growth. Overall, the detection of both DCA and 7-oxo-DCA, in conjunction with the near-complete removal of CA, confirmed 7α-dehydroxylation and 7α-hydroxysteroid dehydrogenase activity, respectively, in both strains.

The c-25 cultures showed a much lower OD_660_ across all conditions, peaking at 0.374 compared to 1.310 and 1.133 for G10 and ATCC 35704^T^, respectively, (Figure 3i–l). The strain exhibited the most rapid growth and highest peak OD_660_ at pH 8. The bile acid profile of c-25 showed a steady decrease in CA with a corresponding increase in DCA and 7-oxo-DCA over the course of 48 h at pH 7 and 8. However, the conversion rate was much slower than that of the *C. scindens* strains, with approximately 70–80% of the CA substrate metabolized by 36 h. The pH 8 condition showed the highest amount of DCA formation, with DCA levels continuing to increase to almost 50% after 36 to 48 h of incubation. Notably, the total bile acid pool also consisted of much higher proportions of 7-oxo-DCA than in the *C. scindens* strains at both pH 7 and 8. This was especially noticeable at pH 7, at which 7-oxo-DCA levels surpassed that of DCA by 36 h. Interestingly, there were no signs of growth or CA conversion at pH 6. At pH 9, although cultures showed clear growth, no DCA production was observed and the only evidence of bile acid transformation was of 7-oxo-DCA formation after 12 h.

In terms of optimal growth conditions, *C. scindens* ATCC 35704^T^, *C. scindens* G10, and *Eubacterium* sp. c-25 appeared to prefer neutral to slightly alkaline conditions, i.e., pH 7 and 8. These were also the conditions that induced the most rapid production of DCA. However, the ability of a DCA producer to 7α-dehydroxylate CA appears to be separate from its ability to grow and proliferate. As shown for the pH 6 and 9 cultures of G10 and ATCC 35704^T^ (Figure 3a,d,e,h), cell density and DCA production are not directly correlated, possibly due to differences in enzymatic activity or induced *bai* gene expression. This is especially evident in c-25, which exhibited higher DCA formation at pH 8 than 7, but no DCA was detected at pH 9 though moderate growth was observed (Figure 3j–l).

### 3.3. Complete Genome Sequencing and Strain Identification

For proper taxonomic identification of c-25 based on phylogenetic analyses using genome sequence information, the complete genome sequence of c-25 was determined along with that of *C. scindens* G10, which also lacked genomic information and would be used in comparative genomic analyses with c-25 (Table 2). Using the newly acquired genomic data, a BLASTn search for the c-25 16S rDNA sequence was conducted to search for closely related species. Interestingly, the closest match was *Lachnoclostridium phocaeense*, a bacterium isolated from human urine [54], at 97.2% sequence identity.

**Figure 4:**
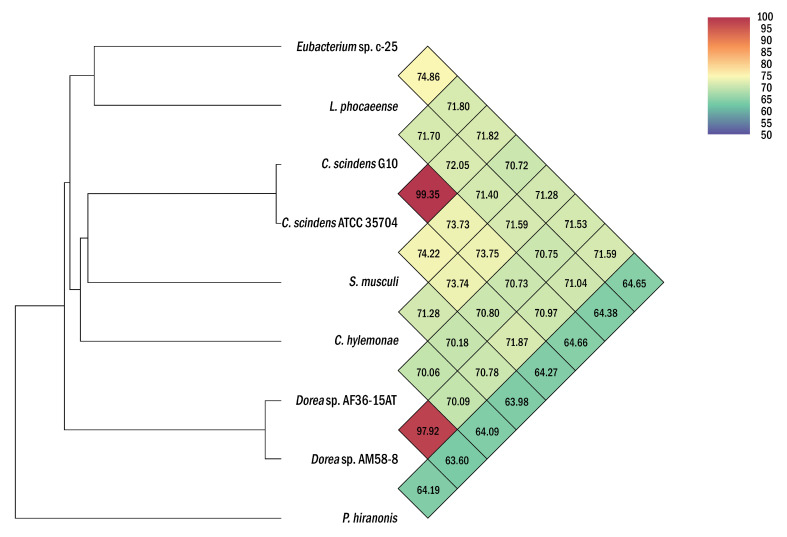
Heatmap of average nucleotide identity (ANI) values between *Eubacterium* sp. c-25, *L. phocaeense*, and other strains relevant to DCA production. Strains listed in Table 1 were used for ANI analysis. Heatmap was generated by OAT [40] and was reconstructed in high-resolution based on the original output.

While this was already below proposed species delineation thresholds of ~98–99% [55,56], ANI analyses were additionally conducted to differentiate c-25 from *L. phocaeense* with more certainty (Figure 4). With results showing only 74.9% ANI, far from the generally accepted 95% intra-species cutoff [57,58], we concluded that c-25 is not the same species as *L. phocaeense*. Moreover, even lower ANI values (64.65% to 71.82%) were observed against the other DCA producers listed in Table 1, demonstrating the phylogenetic uniqueness of c-25 amongst them, at least at the species level. *C. scindens* G10 also displayed similar ANI values to other DCA producers (64.66% to 73.75%) but showed a 99.35% match with *C. scindens* ATCC 35704^T^, confirming its identity as a strain of *C. scindens*.

**Figure 5:**
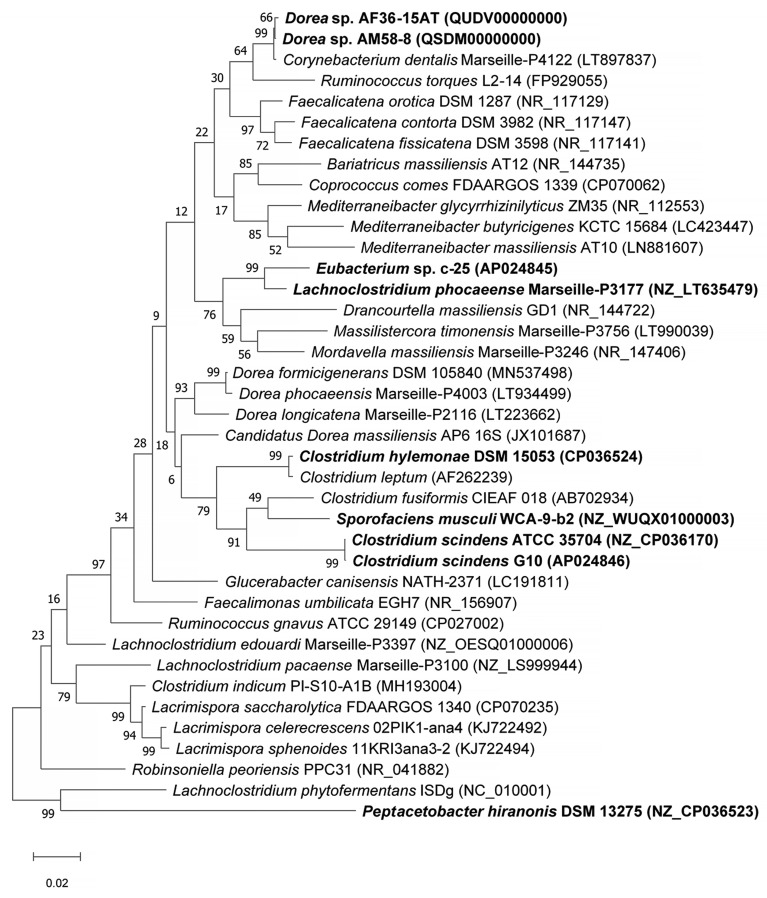
Maximum Likelihood phylogenetic tree of *Eubacterium* sp. c-25 and related species of the intestinal bacteria including known DCA producers. Strains included in the tree construction consisted of the 29 closest 16S rDNA sequence BLASTn matches to *Eubacterium* sp. c-25, excluding uncultured and unidentified strains, and 10 other manually added strains: *L. phocaeense* Marseille-P3177, *C. scindens* G10, *C. scindens* ATCC 35704^T^, *P. hiranonis* DSM 13275, *S. musculi* WCA-9-b2, *Dorea* sp. AF36-15AT, *Dorea* sp. AM 58-8, *Lachnoclostridium edouardi* Marseille-P3397 (NZ_OESQ01000006), *Lachnoclostridium pacaense* Marseille-P3100 (NZ_LS999944), and *Lachnoclostridium phytofermentans* ISDg344 (NC_010001). The strains used in bioinformatics analyses in this study are bolded. The tree was constructed in MEGA X [38] using the Tamura–Nei model [39] with 1000 bootstraps and was rooted at the midpoint. Bootstrap support values are listed next to the branches.

To study the phylogeny and higher-level taxonomy of c-25, a phylogenetic tree was constructed using the top-29 BLASTn hits against the 16S rDNA sequence from c-25, along with those from additional DCA producers: *C. scindens* ATCC 35704^T^, *C. scindens* G10, and *Peptacetobacter hiranonis* (Figure 5). We only included strains with an established or proposed species-level taxonomic designation according to the NCBI Taxonomy Database. Interestingly, *C. hylemonae* was the only DCA producer that appeared within the top 29 hits. The tree also included newly hypothesized DCA producers that were identified later in the study: *Sporofaciens musculi*, *Dorea* sp. AM58-8, and *Dorea* sp. AF36-15AT (Table 1). Finally, because c-25 was most closely related to *L. phocaeense* according to 16S rDNA similarity but no other *Lachnoclostridum* strains were detected within the top BLAST hits, we also added three additional *Lachnoclostridium* strains to predict whether c-25 could be a member of the genus *Lachnoclostridium* by virtue of its phylogenetic distance to other members of this genus: *Lachnoclostridium edouardi* Marseille-P3397 (NZ_OESQ01000006), *Lachnoclostridium pacaense* Marseille-P3100 (NZ_LS999944), and *Lachnoclostridium phytofermentans* ISDg (NC_010001). Thus, a total of 39 strains were included in the phylogenetic tree (Figure 5).

The results indicated that *Eubacterium* sp. c-25 is most closely related to *L. phocaeense* and is distant from other known DCA producers such as *C. scindens*, *C. hylemonae*, and *P. hiranonis*. Unexpectedly, however, the other close relatives do not include additional *Lachnoclostridium* species, but members of the genera *Drancourtella*, *Massilistercora*, and *Mordavella* in the order *Eubacteriales*. Furthermore, other *Lachnoclostridium* species are also not clustered together as would be expected from members of the same genus. These findings indicated that *Eubacterium* sp. c-25 is likely a member of the order *Eubacteriales* but correct classification at family and genus level is not possible due to the ambiguous taxonomic status of the genus *Lachnoclostridium* [59]. Re-examination of their taxonomy and valid publication of taxa names are required, including that of *L. phocaeense*, to obtain reliable taxonomic information on c-25.

### 3.4. Identification of bai Genes

**Figure 6:**
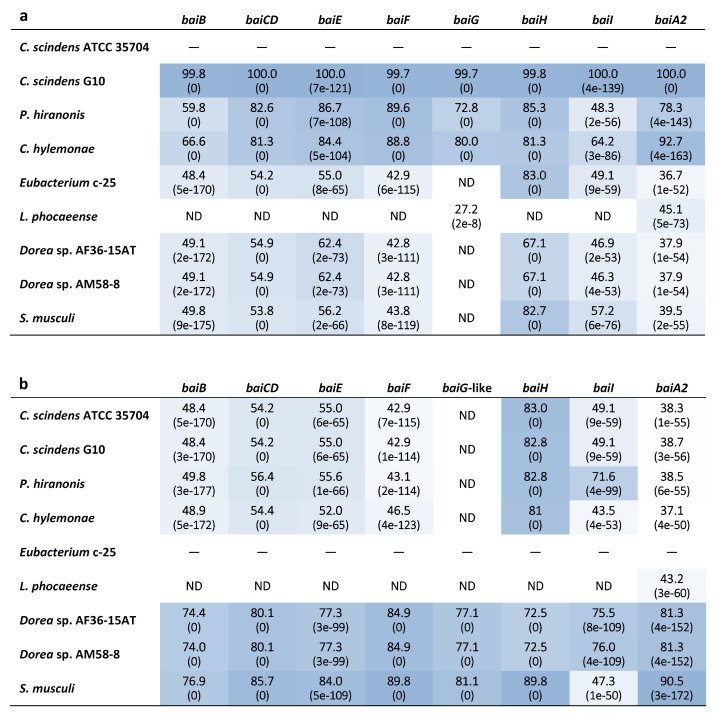
Heatmap table of *bai* gene amino acid sequence identity among known and newly-identified, hypothesized DCA producers. The closest phylogenetic relative of c-25, *L. phocaeense*, was also included. The amino acid sequence identity (%) was evaluated by BLASTp analysis using (**a**) *C. scindens* ATCC 35704^T^ and (**b**) *Eubacterium* sp. c-25 reference *bai* genes as queries. BLAST Expect values (E-values) are given in parentheses. Darker boxes indicate higher degrees of sequence identity. ND = Not detected. See Table S1 for *bai* gene identifier information.

Genome-wide categorization of orthologous genes from c-25 and the other DCA producers using OrthoFinder allowed for the discovery of a set of genes that were predicted to be orthologues of *C. scindens* ATCC 35704^T^ *bai* genes (Supplementary File S1) in both c-25 (EUBC25_24880–EUBC25_24960, EUBC25_02220) and G10 (CSCING10_002180–CSCING10_002270). The protein sequence similarities of predicted c-25 Bai proteins to those of *C. scindens* ATCC 35704^T^ were surprisingly low with the exception of BaiH (Figure 6). A local BLASTp search confirmed that no other proteins encoded in the c-25 genome share greater sequence similarity to *C. scindens* ATCC 35704^T^ Bai proteins than those identified by OrthoFinder, lending support to their predicted identity as *bai* gene orthologues.

**Figure 7:**
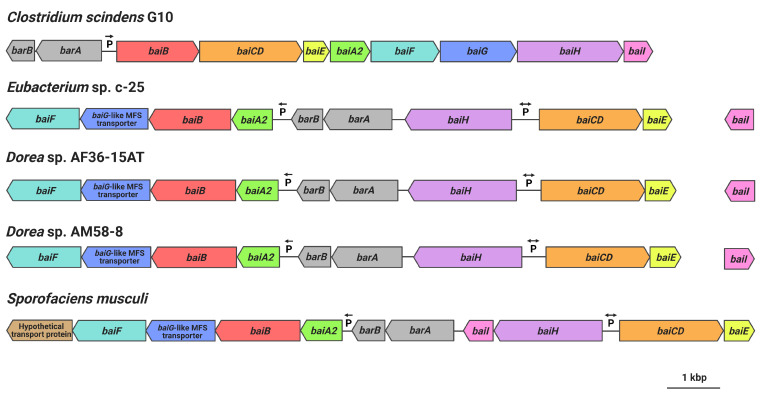
*bai* gene arrangements as observed in *C. scindens* G10, *Eubacterium* sp. c-25, *Dorea* sp. AF36-15AT, *Dorea* sp. AM58-8, and *S. musculi*. Shared genes (or predicted functional homologues in the case of *baiG*) are color-coded. Figure created with BioRender.com.

Gene arrangement of the hypothesized c-25 *bai* genes was found to be very different from that of *C. scindens* (Figure 7). Unlike in *C. scindens* where the entire *bai* gene cluster is controlled by a single promoter and produces a single polycistronic mRNA [60,61], it appears that the *bai* genes of c-25 are separated into multiple gene clusters that are presumably transcribed from different promoter sites (see Table S4 for promoter prediction information). In c-25, *baiA2BGF* and *baiCDE* each constitute a gene cluster, while *baiH* and *baiI* do not share a promoter with other *bai* genes. The genes encoding predicted bile acid-regulatory elements (*barA*, *barB*) proposed to be involved in transcriptional regulation [3] were found upstream of the *baiA2BGF* cluster. In addition, *baiI* is located in a separate chromosomal region far from the rest of the *bai* genes. Importantly, a *baiG* orthologue was not identified in c-25. Instead, a gene encoding a major facilitator superfamily (MFS) transporter similar to the *C. scindens* BaiG transporter existed downstream from *baiB*, though amino acid sequence identity was low (22%, E-value 0.013, BLASTp). We hypothesize that this BaiG-like transporter is a functional homologue of *baiG*.

The same analyses were also performed on the genome of *C. scindens* G10. These analyses identified a *bai* operon structure nearly identical to that of *C. scindens* ATCC 35704^T^ (same gene arrangement as in Figure 1b and ~100% amino acid sequence identity of Bai proteins), as expected of members of the same species. The results confirm that *C. scindens* G10 is included as a conventional 7α-dehydroxylating species both in DCA production profile and the genomic basis for its 7α-dehydroxylation ability.

### 3.5. Expression Analysis of bai Genes in the Presence of CA

As the *bai* gene candidates were successfully identified in *Eubacterium* sp. c-25, we performed qRT-PCR analysis with specific primers for *baiB*, *baiCD*, and *baiH*. The aims of this analysis were to verify the identity of the hypothetical *bai* genes by testing induced expression levels in the presence of CA as observed in *C. scindens* [26] and to compare the expression levels of each gene with those in *C. scindens* G10. It is worth noting that *bai* genes in *C. scindens* ATCC 35704^T^ were shown to be transcribed at a basal level in the absence of CA and could moreover be downregulated to an even lower level of expression [26]. We expected that relative gene expression levels of *baiB*, *baiCD*, and *baiH* would be similar in G10, in which they are transcribed from a single promoter site, while their equivalents in c-25 would show more variance. These *bai* genes were selected because they are representative of the three main *bai* gene clusters in c-25, and were also reported to be essential for 7α-dehydroxylation [14]. In addition, *baiB* is the first gene to be transcribed in the *bai* operon of *C. scindens* while *baiH* is the second-to-last, providing comprehensive coverage of the entire *bai* operon.

**Figure 8:**
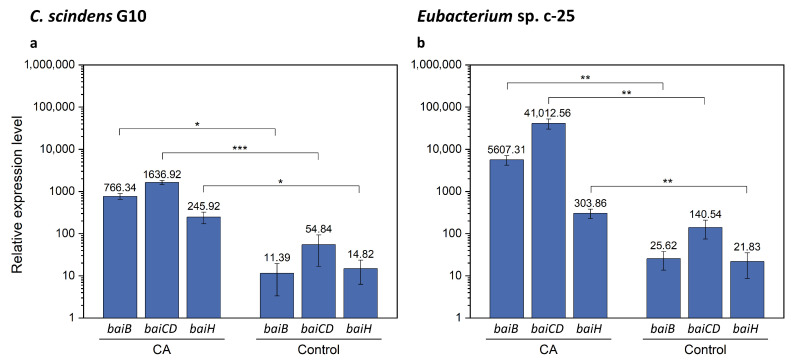
qRT-PCR results showing expression of *baiB*, *baiCD*, and *baiH* genes with or without CA supplementation. Expression was measured in (**a**) *C. scindens* G10 (*n* = 3) and (**b**) *Eubacterium* sp. c-25 (*n* = 7). Cells were cultured in GAM adjusted to pH 8 in the presence (CA) or absence (Control) of 0.1 mM CA and collected for total RNA extraction at mid-exponential growth phase. Mean values of the expression levels of each gene relative to the reference housekeeping gene *recA* are indicated. Error bars denote standard error of the mean. *p*-values lower than 0.05 were considered significant and indicated using numbers of asterisks in the panel (*: *p* < 0.05, **: *p* < 0.01, ***: *p* < 0.001).

The results of qRT-PCR showed that, in the presence of CA, *bai* gene expression in G10 increased by a factor of 67.3, 29.8, and 16.6 for *baiB*, *baiCD*, and *baiH*, respectively (Figure 8a and Table S5). In c-25, expression of these genes increased by a factor of 218.8, 291.8, and 13.9 (Figure 8b and Table S6), suggesting that the predicted *bai* gene orthologues in c-25 are functional *bai* genes. However, there is a considerable difference in gene expression between *bai* genes in c-25, with *baiB* and *baiCD* expressed at levels about 15–20 times higher than *baiH*. In G10, *bai* gene expression levels were relatively similar and decreased according to the downstream order in the operon, as expected.

### 3.6. Identification of Additional 7α-Dehydroxylating Bacteria

Finally, we searched for other bacteria that may possess a similar *bai* gene structure and arrangement as in c-25. Because Bai protein sequences in c-25 were found to be quite different from those of conventional DCA producers, a Microbial Protein BLAST search was conducted using BaiB from c-25. By screening isolated and sequenced species, three bacterial strains were found possessing genes with 74-77% similarity to c-25 BaiB: *Sporofaciens musculi* [62], *Dorea* sp. AF36-15AT [63], and *Dorea* sp. AM58-8 [63]. The presence of other *bai* genes was confirmed in each strain. The arrangement of *bai* genes in these three strains is almost identical to that of c-25 (Figure 7) except that in *S. musculi*, a transporter gene is additionally encoded at the downstream end of the *baiA2-F* operon, and the *baiI* gene is located just downstream of *baiH*. The *baiG*-like transporter found in c-25 was also found in these three new candidate strains in an analogous position (Figure 7).

## 4. Discussion

In this study, the morphology, growth, bile acid metabolism, and genomic information of unique but forgotten secondary bile acid producer *Eubacterium* sp. c-25 were clarified. The list of known intestinal DCA producers has long been limited to a handful of species, with few new isolates being identified over decades of research [19]. To add to this, current models of DCA formation are primarily based on data gathered from *C. scindens* studies. While human fecal isolate *Eubacterium* sp. c-25 has been known to produce DCA [21], the physiological and genomic data newly acquired in this study challenge the current understanding of DCA producers, revealing a unique morphology (Figure 2), dependency on specific pH conditions for optimal growth and 7α-dehydroxylation (Figure 3), and a novel *bai* gene arrangement that could represent an entirely unexplored genotype in DCA producers, as evident by its presence in the multiple, phylogenetically distant intestinal/fecal isolates: *S. musculi*, *Dorea* sp. AF36-15AT, and *Dorea* sp. AM58-8 (Figure 6 and Figure 7).

The filamentous morphology of c-25 has not been observed in other DCA producers. Images of *C. scindens*, *P. hiranonis*, and *C. hylemonae* show that they share a similar rod shape, but any visible end-to-end linkages are limited to two or three-cell chains [18,28,64,65]. The filamentous morphology can be observed in various intestinal bacteria, including species of *Lactobacillus* and *Bifidobacterium* [66,67], but there are few, if any, reports of filamentous *Clostridium* species, as they are predominantly unlinked [66]. This further supports our findings that c-25 is phylogenetically distinct from known DCA producers.

Taxonomically, c-25 appears to be a member of the order *Eubacteriales* as suggested by the results of 16S rDNA-based phylogenetic analysis (Figure 5). However, due to the questionable taxonomic designation of its closest relative *L. phocaeense*, it is difficult to correctly classify c-25 in the current classification scheme of *Lachnoclostridium* [59].

As observed in Figure 3, growth and DCA production of all tested strains, including c-25, were considerably dependent on the medium pH. The idea of a pH range conducive for in vitro growth and 7α-dehydroxylation may hint at certain colonization preferences in the gastrointestinal environment. *C. scindens* appears to be able to proliferate at a relatively wide range of pH conditions, while c-25 may be limited to neutral or slightly alkaline environments. It is possible that the narrow pH range conducive to c-25 growth limits growth of this strain to specific individuals or sections of the gastrointestinal tract exhibiting higher pH. For instance, though results and test methods differed between studies, there seems to be a general consensus that the proximal colon pH ranges from about 5.8–6.7 and rises to 6.1–7.5 in the distal colon and rectum [50]. Gastrointestinal pH increases were also noted in patients with Crohn’s disease and ulcerative colitis [68]. Although the prevalence of c-25 in the human gut is unknown, it is possible that the strain occupies a specific niche such as the distal colon, or is present in higher numbers in irregular physiological conditions causing increased intestinal pH, suggesting that pH can potentially be used as a marker for disease states associated with increased DCA production. Recently, while the link between physiological pH and colonization was not specifically investigated, studies on location-specific colonization of inoculated *C. scindens* in gnotobiotic mouse models were conducted using nanoscale secondary ion mass spectrometry and meta-proteomic analyses [69,70]. The introduction of such advanced technologies and studies specifically tailored to account for the variation in gut pH by location and individual [49,51] are necessary to further investigate the ecological niches of c-25 and other DCA producers.

The requirement for 7α-dehydroxylation has long been the induction of the *baiA2*-*I* gene cluster, controlled by a single promoter and producing a single polycistronic mRNA [60,61]. While a recently published analysis of metagenome-assembled genomes has identified candidate DCA producers possessing various arrangements of the *bai* gene cluster deviating from the arrangement found in *C. scindens* [17], no strains had yet been experimentally proven to produce DCA. The discovery of the uniquely arranged but apparently functional *bai* gene cluster in c-25 and its presence in other bacterial species necessitates a re-examination of DCA producer diversity and the genetic basis for 7α-dehydroxylation. As shown in Figure 8, c-25 displayed considerably increased expression of *baiB* and *baiCD* in the presence of CA. In contrast, the CA-induced increase in *baiH* expression was modest. This disproportionate induction is consistent with the fact that c-25 *bai* genes are divided into multiple, separately transcribed gene clusters, but raises the question of how *bai* gene expression is both coordinated and regulated in c-25. The physiological and metabolic impact of these highly expressed *bai* genes requires further study, which may yield insights into the factors that influence DCA formation in the gastrointestinal environment.

The comparative analysis of *bai* genes in c-25 and *C. scindens* led to several questions regarding their function and conservation. First, although we hypothesize that the BaiG-like protein is a functional homologue of the bile acid transporter BaiG [71], this has yet to be confirmed experimentally. As it shares only 22% amino acid sequence identity with the BaiG protein in *C. scindens*, there are likely to be considerable differences in protein structure and biological functionality. Secondly, *baiI*, thought to encode a 7β-dehydratase [3] involved in the 7β-dehydroxylation of ursocholic acid (epimer of CA possessing a 7β-hydroxy group) to DCA, is found within the *bai* operon in previously analyzed DCA producers, but this gene is not co-localized with other *bai* genes in c-25 or the two *Dorea* species that share the same *bai* gene arrangement as c-25. As *baiI* has actually been shown to be unnecessary for 7α-dehydroxylation in vitro [14], there is a possibility that *baiI* is differentially expressed in c-25 and the two *Dorea* species only when substrates possessing the 7β-hydroxy group are available. Thirdly, in contrast to the other *bai* genes, *baiH* in c-25 showed high similarity to *baiH* from *C. scindens* (Figure 6), suggesting that it has an essential role in 7α-dehydroxylation and is an unsubstitutable protein. As noted in a recent study, protein sequences of *baiH* and its homologue *baiCD* appear to be more highly conserved than the other *bai* genes among homologues identified in metagenomic datasets [72], though *baiCD* sequence identity was not considerably high for c-25 in this case. As for the rest of the *bai* genes in c-25, all exhibited surprisingly low protein sequence similarity, with percent identity at ~50% (Figure 6). Due to the complexity of biochemical modifications required at each step of the 7α-dehydroxylation enzyme cascade [14], we expected a higher degree of *bai* gene conservation among DCA producers. However, the low protein similarity observed between c-25 and *C. scindens*, along with varying levels of differential expression presumably resulting from different gene arrangements, suggest that there may be more genotypic variability among DCA producers than previously expected.

The observation that the unique arrangement of *bai* genes found in *Eubacterium* sp. c-25 were found in other bacteria (i.e., *S. musculis* and *Dorea* spp.) is strong evidence for the existence of alternative *bai* genotypes in intestinal DCA producers. Large-scale metagenomic analyses suggest that *bai* genes are much more common in intestinal microbial communities than previously believed [17,72], calling into question the actual numbers and diversity of DCA producers beyond the few identified thus far [15,19].

## 5. Conclusions

The physiological and comparative genomic analyses of *Eubacterium* sp. c-25 conducted in this study demonstrate that this strain is not a typical DCA producer when compared to known DCA producers. Further investigation, including functional analysis of predicted *bai* genes and quantification of c-25 and other strains sharing similarly organized *bai* genes in gut microbial populations, can reveal the extent to which these strains contribute to DCA formation in the human intestines. Understanding the factors that drive DCA production is essential for understanding gastrointestinal health in a biomedical context due to DCA’s connection to diseases such as liver and colon cancer [6,7,8,9] and cholesterol gallstone disease [10,11]. As an example, dietary prebiotic supplementation has been shown to decrease cecal DCA levels in rats [73], suggesting that a similar approach may be beneficial in treating humans exhibiting disease states correlated with abnormal DCA levels. However, the development of such treatments and their safe application to humans requires a thorough understanding of bile acid metabolism, key DCA producers, and host-microbe interplay. This study provides novel insights into these areas in the hopes of elucidating the holistic role of DCA producers in the human gut and their impact on gastrointestinal health.

## Figures and Tables

**Table 1:** Genome information of the strains used in bioinformatics analyses.

Organism	Genome Size (bp)	Database	Accession No.	Source
*Eubacterium* sp. c-25	3,042,110	DDBJ	AP024845	Human
*Clostridium scindens* G10	3,315,593	DDBJ	AP024846	Rat
*Clostridium scindens* ATCC 35704^T^	3,658,040	NCBI RefSeq	NZ_CP036170	Human
*Clostridium hylemonae* BSD2780061688st1_A6	3,793,913	NCBI RefSeq	NZ_SPFR01000000	Human
*Peptacetobacter hiranonis* DSM 13275	2,521,899	NCBI RefSeq	NZ_CP036523	Human
*Lachnoclostridium phocaeense* Marseille-P3177	3,500,754	NCBI RefSeq	NZ_LT635479	Human
*Sporofaciens musculi* WCA-9-b2	5,763,728	NCBI RefSeq	NZ_WUQX01000000	Mouse
*Dorea* sp. AF36-15AT	2,953,222	JGI IMG	2840399886 ^1^	Human
*Dorea* sp. AM58-8	3,102,867	JGI IMG	2854065680 ^1^	Human

^1^ IMG taxon ID.

**Table 2:** Complete sequenced genome information.

	*Eubacterium* sp. c-25	*C. scindens* G10
Genome size (bp)	3,042,110	3,315,593
GC content (%)	44.5	46.5
No. of CDSs	2893	3292
Average Protein Length	315.6	297.7
Coding Ratio (%)	90.0	88.7
No. of rRNAs	8	12
No. of tRNAs	55	58

## Data Availability

Whole-genome sequences of *Eubacterium* sp. c-25 and *Clostridium scindens* G10 are publically available in the Genbank/EMBL/DDBJ database under the accession numbers AP024845 and AP024846, respectively.

## Supplementary Materials

The following are available online at https://www.mdpi.com/article/10.3390/microorganisms9112254/s1, Table S1: *bai* gene identifiers by locus tag, Table S2: qRT-PCR primer sequences, Table S3: Initial and final medium pH in *Eubacterium* sp. c-25, *C. scindens* G10, and *C. scindens* ATCC 35704^T^ cultures, Table S4: Predicted *bai* gene promoter regions, Table S5: *C. scindens* G10 *bai* gene qRT-PCR data, Table S6: *Eubacterium* sp. c-25 *bai* gene qRT-PCR data, Supplementary File S1: OrthoFinder raw data.
