# Supplementary material for: Comparative Genomic and Physiological Analysis against Clostridium scindens Reveals Eubacterium sp. c-25 as an Atypical Deoxycholic Acid Producer of the Human Gut Microbiota
Source: Microorganisms. 2021 Oct 29;9(11):2254. doi: 10.3390/microorganisms9112254 (PMC8623032; doi:10.3390/microorganisms9112254)
Supplement: Supplementary file 1 [file microorganisms-09-02254-s001.zip › Supplementary Tables.pdf]

Table S1. *bai* gene identifiers by locus tag.

	baiA2	***baiB***	***baiCD***	***baiE***	***baiF***	***baiG***	***baiG*-like**	***baiH***	***baiI***
***Eubacterium* sp. c-25**	EUBC25_24910	EUBC25_24900	EUBC25_24950	EUBC25_24960	EUBC25_24880	ND	EUBC25_24890	EUBC25_24940	EUBC25_02220
***C. scindens* G10**	CSCING10_002230	CSCING10_002200	CSCING10_002210	CSCING10_002220	CSCING10_002240	CSCING10_002250	ND	CSCING10_002260	CSCING10_002270
***C. scindens* ATCC 35704**	HDCHBGLK_RS12450	HDCHBGLK_RS07215	HDCHBGLK_RS07220	HDCHBGLK_RS07225	HDCHBGLK_RS07235	HDCHBGLK_RS07240	ND	HDCHBGLK_RS07245	HDCHBGLK_RS07250
C. hylemonae	FGQ83_RS08750	FGQ83_RS09845	FGQ83_RS09840	FGQ83_RS09835	FGQ83_RS08760	FGQ83_RS09825	ND	FGQ83_RS09820	FGQ83_RS09815
P. hiranonis	KGNDJEFE_RS02005	KGNDJEFE_RS01990	KGNDJEFE_RS01995	KGNDJEFE_RS02000	KGNDJEFE_RS02010	KGNDJEFE_RS02015	ND	KGNDJEFE_RS02020	KGNDJEFE_RS01975
L. phocaeense	BQ7370_RS13730	ND	ND	ND	ND	BQ7370_RS08465	ND	ND	ND
S. musculis	GN277_RS03065	GN277_RS03060	GN277_RS03090	GN277_RS03095	GN277_RS03050	ND	GN277_RS03055	GN277_RS03085	GN277_RS03080
***Dorea* sp. AF36-15AT**	Ga0338879_1972	Ga0338879_1971	Ga0338879_1976	Ga0338879_1977	Ga0338879_1969	ND	Ga0338879_1970	Ga0338879_1975	Ga0338879_2651
***Dorea* sp. AM58-8**	Ga0337498_45_28937_29713	Ga0337498_45_29756_31321	Ga0337498_45_22091_24013	Ga0337498_45_21528_22088	Ga0337498_45_32640_34004	ND	Ga0337498_45_31311_32591	Ga0337498_45_24473_26467	Ga0337498_17_14866_15417

Table S2. qRT-PCR primer sequences.

Strain	Target	Primer length (bp)	GC%	Tm (°C)	Product length (bp)	Primer sequence (5' to 3')
***Eubacterium* sp. c-25**	recA (Fw)	20	55	60.04	78	ACGGAGACATTGAAGCAGGG
	recA (Rv)	20	50	60.04	78	AGGCGCGATCTTGTTCTTCA
	baiB (Fw)	20	55	60.04	72	GCGGAACGTCAGGAAAAACC
	baiB (Rv)	20	55	60.04	72	CCCGAAGCCCTTCATCACTT
	baiCD (Fw)	20	50	60.04	133	TTCACGGAGCGCACACTTAT
	baiCD (Rv)	20	55	59.97	133	GCACGCATTTCCCGGATTAC
	baiH (Fw)	20	55	60.04	98	GAGGCAGGCAGTGTTCTGAT
	baiH (Rv)	20	55	60.04	98	ACATTGGCAGAAGGTCCGAG
***C. scindens* G10**	recA (Fw)	20	50	59.9	124	ATAAGATCGCGCCTCCGTTT
	recA (Rv)	20	55	60.04	124	CAGGCTCCGCTTTTGTTGAC
	baiB (Fw)	20	55	60.11	188	AGATGGTGCCGACCTTGATG
	baiB (Rv)	20	50	60.04	188	AAGGCCGATGCATTCAGTCA
	baiCD (Fw)	20	55	59.96	129	AGCAGGGCAAGGTTCACTAC
	baiCD (Rv)	20	50	59.96	129	TTGCCTTCAGTCCATCGCTT
	baiH (Fw)	20	50	59.97	110	TGCCCGTTTTGTGATCGAGA
	baiH (Rv)	20	55	60.03	110	GGCTGTTGCCTCCTACCATT

Table S3. Initial and final medium pH in *Eubacterium* sp. c-25, *C. scindens* ATCC 35704, and *C. scindens* G10 cultures.

Strain	Initial pH target	Initial pH (0h)[1]	Final pH (48h)[2]
***Eubacterium* sp. c-25**	6	6.03	6.04
	7	7.03	6.71
	8	7.90	7.34
	9	8.86	8.37
***C. scindens* G10**	6	6.02	5.85
	7	7.11	6.31
	8	7.98	6.72
	9	8.96	7.35
***C. scindens* ATCC 35704**	6	5.97	5.53
	7	7.09	6.33
	8	7.89	6.71
	9	8.94	7.50

[1] All biological replicates inoculated using same medium source
[2] Mean, *n* = 3

Table S4. Predicted *bai* gene promoter regions.

Organism	Downstream gene	Predicted -35 to -10 sequence	-35 sequence	-10 sequence	LDF[1]
***Eubacterium* sp. c-25**	**baiA2**	TTGTAAAAAGAGTCATTGTTAAAAATATATT	TTGTAA	AAATATATT	6.8
	**baiCD**	TCGAAATTTTGTACATTGTTTGTATATAAT	TCGAAA	GTATATAAT	6.76
	**baiH**	TTCCTGCAGGAGCAAAAATTTGATTATTAT	TTCCTG	GATTATTAT	6.37
***C. scindens* G10**	**baiB**	CTGAAAATTATGTGAGAAGTTACAAT	CTGAAA	AGTTACAAT	5.13
S. musculi	**baiA2**	TTTATTTGTCCGTCATTTTGACTATATATT	TTTATT	CTATATATT	7.73
	**baiCD**	TTGATTTTTTGTACATTGTTTGTGCATTATAAT	TTGATT	CATTATAAT	6.69
	**baiH**	GTGACGCGCCCCCTTTTTTATTCGTTTATAAT	GTGACG	GTTTATAAT	9.88
***Dorea* sp. AF36-15AT**	**baiA2**	TTTAAAGCCTGGTTTTGCTGTTAAAAT	TTTAAA	TGTTAAAAT	6.02
	**baiCD**	TTAATTAGAAAAAATGTGATAGGAAAAAT	TTAATT	AGGAAAAAT	5.8
	**baiH**	TTTCTAATTAATCATTTTTTCTTTTTTATGCT	TTTCTA	TTTTATGCT	8.71
***Dorea* sp. AM58-8**	**baiA2**	TTTAAAGCCTGGTTTTGCTGTTAAAAT	TTTAAA	TGTTAAAAT	6.02
	**baiCD**	TTAATTAGAAAAAATGTGATAGGAAAAAT	TTAATT	AGGAAAAAT	5.8
	**baiH**	TTTCTAATTAATCATTTTTTCTTTTTTATGCT	TTTCTA	TTTTATGCT	8.71

[1] Linear discriminant function score for promoter prediction based on threshold value of 0.20

Table S5. *C. scindens* G10 *bai* gene qRT-PCR data.

Gene	Condition	Rep. 1 relative expression	Rep. 2 relative expression	Rep. 3 relative expression	Mean	SEM	Mean fold-increase	*t*-test	*p*-value
baiB	**CA**	539.951477	819.61972	939.44026	766.3372	118.3598	67.26357103	Welch	0.01164
baiB	**Control**	0.86838821	6.0405947	27.270164	11.39305	8.077747			
baiCD	**CA**	1331.10576	1618.5573	1961.1067	1636.923	182.0973	29.8463955	Student	0.0005246
baiCD	**Control**	9.75196282	23.973941	130.80886	54.84492	38.20321			
baiH	**CA**	139.84117	392.52117	205.38468	245.9157	75.70528	16.5880209	Welch	0.04541
baiH	**Control**	1.98030046	11.383643	31.110743	14.8249	8.58346			

Table S6. *Eubacterium* sp. c-25 *bai* gene qRT-PCR data.

Gene	Condition	Rep. 1 relative expression	Rep. 2 relative expression	Rep. 3 relative expression	Rep. 4 relative expression	Rep. 5 relative expression	Rep. 6 relative expression	Rep. 7 relative expression	Mean	SEM	Mean fold-increase	*t*-test	*p*-value
baiB	**CA**	2627.50937	3843.2322	10529.588	5065.1703	1732.5276	4165.6933	11287.469	5607.3129	1429.8712	218.825003	Welch	0.003975
baiB	**Control**	6.56020773	89.505772	13.294219	10.864209	5.8752632	6.4192499	46.853589	25.624644	11.975883			
baiCD	**CA**	16271.6429	37287	55626.805	25488.79	18311.081	34253.808	99848.815	41012.563	11023.027	291.816998	Welch	0.004997
baiCD	**Control**	47.3531464	391.94523	30.610429	53.302075	27.921775	28.073646	404.58814	140.54206	66.661246			
baiH	**CA**	164.510447	221.32272	601.66895	203.81953	150.21747	202.01823	583.43753	303.85641	75.130099	13.9177951	Welch	0.004546
baiH	**Control**	6.16941653	35.979996	2.7941747	5.734937	3.0317314	2.7754025	96.339905	21.832223	13.215313			
